# The Incidence of Sexually Dimorphic Gene Expression Varies Greatly between Tissues in the Rat

**DOI:** 10.1371/journal.pone.0115792

**Published:** 2014-12-30

**Authors:** Russell D. J. Huby, Philip Glaves, Richard Jackson

**Affiliations:** Department Molecular Toxicology, Global Safety Assessment, AstraZeneca, Macclesfield, Cheshire, United Kingdom; Centro Cardiologico Monzino IRCCS, Italy

## Abstract

The sexually dimorphic expression of genes across 26 somatic rat tissues was using Affymetrix RAE-230 genechips. We considered probesets to be sexually dimorphically expressed (SDE) if they were measurably expressed above background in at least one sex, there was at least a two-fold difference in expression (dimorphism) between the sexes, and the differences were statistically significant after correcting for false discovery. 14.5% of expressed probesets were SDE in at least one tissue, with higher expression nearly twice as prevalent in males compared to females. Most were SDE in a single tissue. Surprisingly, nearly half of the probesets that were (SDE) in multiple tissues were oppositely sex biased in different tissues, and most SDE probesets were also expressed without sex bias in other tissues. Two genes were widely SDE: Xist (female-only) and Eif2s3y (male-only). The frequency of SDE probesets varied widely between tissues, and was highest in the duodenum (6.2%), whilst less than 0.05% in over half of the surveyed tissues. The occurrence of SDE probesets was not strongly correlated between tissues. Within individual tissues, however, relational networks of SDE genes were identified. In the liver, networks relating to differential metabolism between the sexes were seen. The estrogen receptor was implicated in differential gene expression in the duodenum. To conclude, sexually dimorphic gene expression is common, but highly tissue-dependent. Sexually dimorphic gene expression may provide insights into mechanisms underlying phenotypic sex differences. Online data are provided as a resource for further analyses (GEO reference GSE63362).

## Introduction

Many genes are known to be sexually dimorphically expressed (SDE). Several have been identified though hypothesis-driven investigation, providing a rationale for phenotypic differences between the sexes. For example, male rats are more susceptible to spontaneous hypersensitivity, an effect attributed to the androgen-mediated induction of angiotensinogen in the male kidney [1]. Others have been discovered by serendipity, with sexually dimorphic expression being observed in the course of characterizing newly identified genes. In some instances the sexually dimorphic expression of genes can be rationalized as relevant to functional differences between the sexes, though often its relevance is obscure.

With global transcript profiling, it is possible to identify essentially all SDE genes within a tissue, as has been carried out in the liver [2], heart [3], [4] and brain [5]. This enables a tissue-centric analysis of SDE genes to be conducted, but does not allow gene-centric analysis, i.e. the expression patterns of those same genes in other tissues is not considered. Gene expression profiling across multiple tissues from both sexes in the rat has been conducted before, though only four somatic tissues were included [6].

Here, we carried out gene expression profiling of 26 somatic and 5 sex-specific tissues from both sexes of the rat on Affymetrix RAE-230v2 Genechips. The resulting data set enabled us to characterize the expression of SDE genes across a wide range of rat tissues affording new insights into the distribution of SDE genes that may have functional implications. Here, we focus on the patterns of SDE probeset expression that can be discerned from the data. We also consider, by way of example, the expression of specific genes across tissues, and the relationships between SDE genes within specific tissues. Many further analyses are possible from this dataset. The raw and derived data are therefore provided as a resource (S1 Table and GEO GSE63362).

## Methods

### Animals

Five male and five female Han Wistar-derived rats (AlpkHsdBrlHan:WIST) were housed in groups of 2–3. In accordance with the United Kingdom Animal (Scientific Procedures) Act of 1986, this study did not require a Home Office project license because no regulated procedures were carried out. Rats were humanely killed at a designated establishment by halothane inhalation, which is an appropriate method under Schedule 1 of the Act.”Organs and tissues were removed and cleaned of extraneous tissue by a 5-person necropsy team within 5 min of death, then frozen in liquid nitrogen. Pancreas was prioritized for rapid freezing, since its mRNA is known to be particularly vulnerable to degredation. Tissue samples from each animal weighing 100–250 mg were taken from frozen samples and homogenized immediately in RNeasy lysis buffer. Care was taken to ensure samples were taken consistently from the same region of tissue for each individual, and that they were representative of the whole organ (e.g. radial segments of kidney were taken from the equator in each case).

### Gene expression profiling

26 somatic tissues from 5 male and 5 female rats were expression-profiled using Affymetrix RAE230_2 rat genechips. Sex-specific tissues (testis, epididemis, uterus and ovary) were also profiled. Summary data are provided in S1 Table.

RNA was purified from lysates using RNeasy mini columns according to the manufacturer’s instructions (Qiagen Inc, CA.). Nucleic acid (RNA) purity was determined according to the 260/280 nM absorbance ratio using a Nanodrop-1000 (Thermo Scientific). Samples were accepted only if the ratio fell between 1.8 and 2.2. Otherwise, RNA was re-extracted from new samples of frozen tissue. RNA integrity was assessed using the 2100 Bioanalyser (Agilent). Samples with an RNA integrity number (RIN) <7 were rejected, and re-processed. cDNA, cRNA and fragmented hybridization probes were prepared using the Affymetrix IVT kit (Affymetrix CA.). Only samples meeting all quality criteria were progressed. When samples failed any of these criteria, new preparations from frozen tissue were substituted. Hybridization mixes were prepared and applied to Affymetrix RAE230_2 rat genechips. After hybridization, genechips were washed and scanned using an Affymetrix genechip scanner according to the manufacturer’s instructions.

### Data QC

Chip quality control was assessed using the AffyQCMetrics package in Bioconductor, an open source bioinformatics suite. Individual chips were examined to ensure they met quality standards in all of the tests performed (the test for actin 3′/5′ ratio was excluded) (see S2 Table). This led us to eliminate at most two chips per organ per sex, mainly due to indications that RNA had degraded.

### Statistical and pathway analysis

Signal strength measurement and normalisation was determined using the Gene Chip Robust Multichip Average (GC-RMA) algorithm (Bioconductor). GCRMA normalisation performs background subtraction using information on the probe sequence composition through the calculation of binding affinities[7]. No additional normalization is required. Statistical differences between male and female expression values were determined using Student’s 2-tailed T-test, then applying the Benjamin-Hochberg correction for false discovery.

Probesets were considered to be SDE in a given tissue if all of the following criteria were met: 1) The mean Robust Multichip Average (RMA-S) expression value exceeded 6.65 in at least one sex. This was to exclude probesets that were not measurably expressed above background noise levels. 2) At least a 2-fold difference in expression existed between sexes (log2 expression difference >+/−1). This limited analysis to genes that were differences in expression were likely to have a significant biological consequence. 3) The difference in expression between the sexes had an FDR q-value of less than 0.05 (Benjamin-Hochberg FDR correction).

Hierachical clustering using the Ward method was carried out in JMP (SAS, NC).

Gene network analysis was conducted using Ingenuity Pathway Analysis (IPA) software (Ingenuity Systems Inc, CA.).

The RAE_230_2 genechip includes a number of probesets that have not been mapped to a specific gene. In some instances, multiple probesets may map to a single gene. It is not therefore always possible to equate probesets with genes. Where mapping is unambiquous, the term gene is employed; otherwise, the term probeset is used.

## Results

### Incidence of SDE probesets

Approximately 61% (19,007 out of 31,042) of all probesets on the RAE230_2 genechip were measurably expressed above background (mean RMA-S value >6.65) in at least one tissue, one sex (Table 1). 14.5% of these (2771 out of 19,007) were SDE in at least one tissue; 87% of these in turn (2422 out of 2771) were SDE in a single tissue.

**Table 1:** The distribution of SDE probesets across rat tissues.

Probeset property:	Always male biased	Always female biased	Oppositely biased in different tissues	Total
When sex biased∧∧:				
Probesets present on chip*				31042
**Expressed in at least 1 tissue**				19007
**SDE in at least 1 tissue**	1704	901	166	2771
**No. of tissues in which probesets are SDE:**				
** 1**	1573	849	N/A	2422
** 2**	115	43	132	290
** 3**	14	4	30	48
** 4**	1	0	4	5
** 19**	0	2	0	2
** 20**	0	1	0	1
** 22**	0	2	0	2
** 24**	1	0	0	1

∧∧ probesets expressed with sex bias in one or more tissues may be expressed without bias in others.

The remaining 13% of SDE probesets (349 out of 2747) were SDE in multiple (2–24) somatic tissues. The frequency of SDE probesets in multiple tissues dropped away rapidly for increasing numbers of tissues. Surprisingly, 47.6% of probesets SDE in multiple tissues (166 out of 349) were oppositely sex-biased in different tissues.

Most (93%) SDE probesets were also expressed without sex bias in other tissues.

More SDE probesets were preferentially expressed in males vs females (1704 *vs* 901). For probesets that were SDE in multiple tissues, the difference was 131 *vs* 52. By contrast to somatic tissues, 69.1% of the probesets expressed in either the testes and ovary were differentially expressed between these organs.

### SDE probesets have distinct characteristics

26% of all expressed non-SDE probesets were expressed in all tissues, compared to only 9% of SDE probesets (without dimorphism in most tissues). This difference seems sensible, since many ubiquitous genes carry out housekeeping activities, equally necessary in both sexes, all tissues.

15% of all non-SDE probesets were expressed in a single tissue, compared to only 8% of SDE probesets. This difference is harder to rationalize, but together these observations confirm that, as a population, SDE probesets are distinct from non-SDE probesets. Together, these observations indicate that SDE probesets represent a discrete subset of genes with a distinct distribution and distinct functional characteristics.

### Identification of genes with ubiquitous sexually dimorphic expression

Only six probesets were SDE in a majority of tissues (i.e. more than 13 out of 26). They stood out from other probesets that were SDE in a maximum of 4 tissues, since they were SDE in a minimum of 19 tissues. All six probesets showed consistent sex bias (i.e. were biased in the same direction in all tissues), and all showed the same direction of bias even in tissues where they failed to meet all threshold criteria applied in this analysis; in effect, they were ubiquitously SDE.

**Figure 1:**
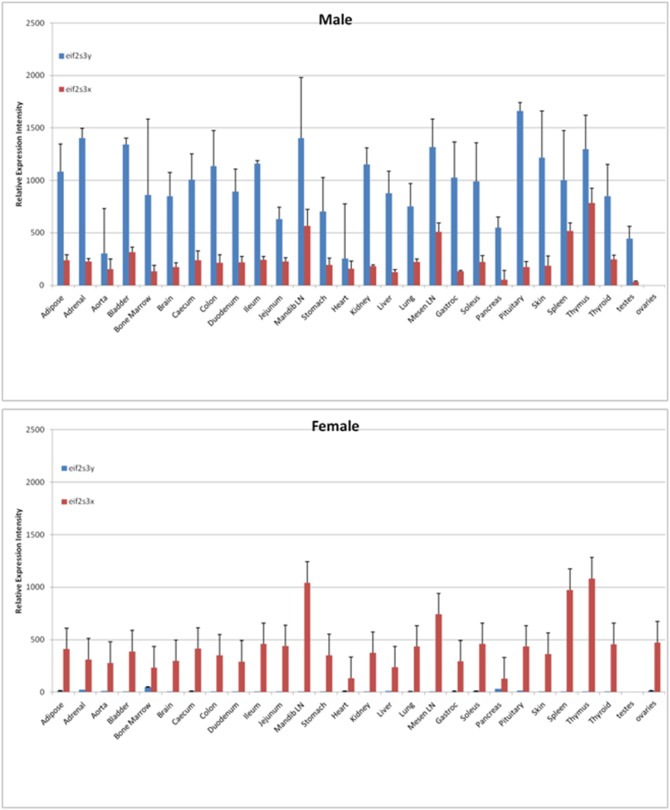
Expression of *Eif2s3x* and *Eif2s3y* in male and female rat tissues. Expression level is relative to the mean expression of the probeset across all tissues both sexes. Error bars represent standard deviations.

Five of these probesets were expressed preferentially (essentially exclusively) in females, and all mapped to the *Xist* gene, which is involved in the inactivation of the X chromosome [8]. The sixth probeset was expressed preferentially (essentially exclusively) in males, and mapped to the gene *Eif2s3y*, a eukaryotic translation initiation factor subunit encoded on the Y chromosome. *Eif2s3x*, a homologue on the X chromosome [9], was significantly more highly expressed in four tissues in the female, with a trend towards higher female expression in all tissues, averaging 1.7-fold (p-value for difference between male and female expression of Eif2S3x for all tissues <0.0001) (Fig. 1).

### Hierarchical clustering of SDE probesets

**Figure 2:**
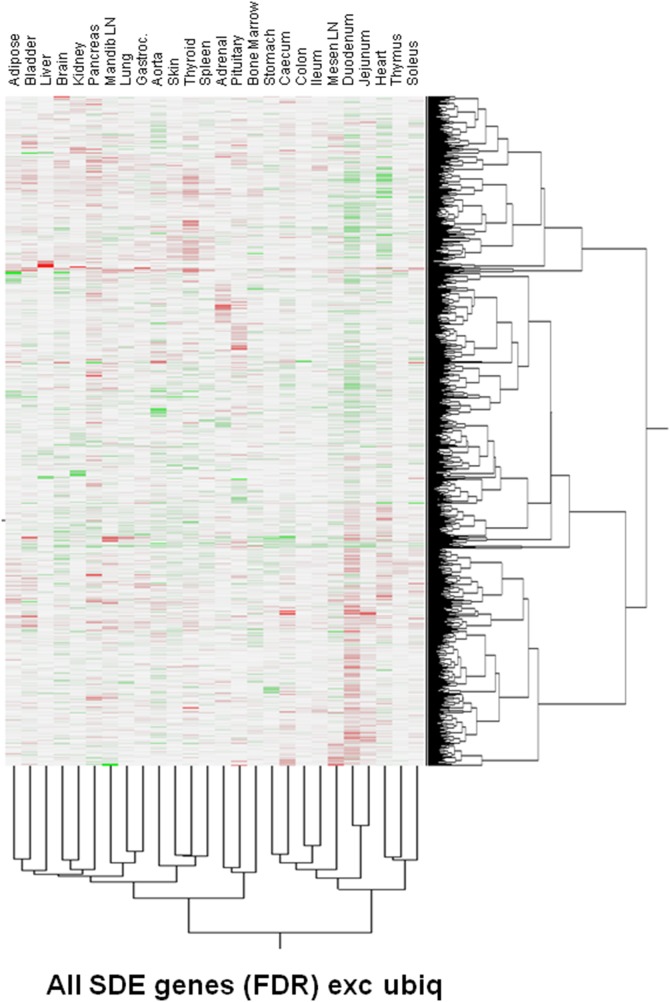
2-way hierarchical clustering of probesets that are SDE in 1–4 tissues. Exc. Ubiq. denotes that the six probesets SDE in 19+ tissues are excluded, as they unduly dominate the clustering of probesets. Colour intensity reflects the –fold difference in expression between males and females: red = more highly expressed in females, blue  =  more highly expressed in males. Colour intensity is globally normalized across all tissues and all included probesets.

Two-way hierarchical clustering of the male:female expression ratios of SDE probesets was carried out (Fig. 2). Only weak clustering of tissues was seen, with the greatest similarity observed between the duodenum and jejunum. Tissues were weakly divided into two large groups, with the GI tract and mesenteric lymph node, heart thymus and soleus in one, and the remaining tissues in the other.

Three major clusters of probesets were identified; group 1 consisted of probesets that were broadly male-selective in the GI tract group and female-selective in the other tissue group; group 2 was broadly male-selective, and group 3 female selective. Within all groups, however, complex combinations of male- and female- selective expression across tissues was evident.

We speculated that clusters of SDE genes may occur in multiple tissues that could not be readily observed by hierarchical clustering. We therefore determined the pattern of SDE probeset sharing between tissues. All occurrences are recorded in Table 2 (region above the diagonal). We then determined the level of enrichment of shared probesets between tissues, i.e. the degree to which the incidence of shared probesets exceeded that which would be expected by chance, given the frequency of SDE probesets in each tisssue (region below the diagonal). 101 probesets were common between the duodenum and kidney with an enrichment factor of 1.3, equating to 30 more probesets than would be expected by chance. 31 probesets were common between the adrenal and pituitary with an enrichment factor of 1.8, equating to 14 more probesets than might be expected by chance. Other commonalities were considered too small to be distinguishable from noise. Overall, it appears that there is little commonality in sexually dimorphic gene expression between tissues.

**Table 2:** The incidence of SDE probesets common to pairs of tissues.

	Duodenum	Kidney	Pituitary	Liver	Adrenal	Skin	Adipose	Aorta	Jejunum	Ileum	Pancreas	Bladder	Caecum	Colon
Duodenum	**214**	101	42	46	31	8	11	6	8	1	1	0	0	0
Kidney	1.3	**175**	30	38	21	13	3	1	1	3	0	1	0	0
Pituitary	0.9	0.8	**99**	13	31	3	6	6	0	0	0	0	0	0
Liver	1.1	1.1	0.7	**93**	14	2	5	2	0	0	0	0	0	0
Adrenal	0.8	0.7	1.8	0.9	**82**	5	2	1	1	0	0	0	0	0
Skin	0.6	1.3	0.5	0.4	1	**27**	1	0	0	0	0	0	0	0
Adipose	1	0.3	1.1	1	0.5		**25**	0	0	0	1	0	0	0
Aorta	1		2.2					**13**	0	0	0	0	0	0
Jejunum	2.2								**8**		0	0	0	0
Ileum		2								**4**	0	0	0	0
Pancreas											**1**	0	0	0
Bladder												**1**	0	0
Caecum													**1**	1
Colon														**1**

The number of SDE probesets common to every pair of tissues (regardless of the direction of sex bias) was determined (figures above diagonal). The degree of enrichment in common SDE probesets, compared to what might be expected at random, is shown below the diagonal. This is defined as: actual number of SDE probesets in common/(fraction of SDE probesets in tissue A * fraction of SDE probesets in tissue B * total number of probeset pairs). For probesets that are SDE in 2 tissues, the number of pairs is 1; for 3, 3, and for 4, 6.

### Tissue distribution of SDE probesets

The number of SDE probesets varied widely between tissues (Table 3). The greatest number was seen in the duodenum (1176), with none (other than the ubiquitous Eif2s3y and Xist probesets) seen in 12 out of 26 surveyed tissues. Although the precise figures are sensitive to the statistical methods used and the thresholds applied, the wide disparity across the range of tissues seen in Table 1 was robust. The duodenum, kidney, adrenal, jejunum and ileum showed a clear preponderance of male-biased SDE probesets, whilst the adipose alone had a preponderance of female-biased SDE probesets. Similar numbers of each were seen in the pituitary, liver, skin and aorta.

**Table 3:** The incidence of SDE probesets by tissue.

	#SDE probesets that are expressed in one tissue, one sex		#SDE probesets that are ubiquitous in other tissues*		Total #SDE probesets*	
	Higher in:		Higher in:		Higher in:	
Tissue	M	F	M	F	M	F
Duodenum	8	1	79	39	896	274
Kidney	26	29	58	24	424	260
Pituitary	12	21	14	16	221	203
Adrenal	19	6	23	10	234	112
Liver	18	15	14	14	164	160
Adipose	0	1	0	6	27	65
Skin	2	3	5	1	31	30
Aorta	0	0	0	1	18	19
Jejunum	0	0	2	0	11	0
Ileum	0	0	2	0	11	0
Pancreas	0	0	0	0	0	2
Bladder	0	0	0	0	1	1
Caecum	0	0	0	0	1	0
Colon	0	0	0	0	1	0

Tissues are ranked according to the total number of SDE probesets they express.

*Six probesets corresponding to *Eif2s3y* and *Xist* are excluded.

There were no SDE probesets (other than the six excluded) in the following tissues: Thyroid, Gastrocnemius, Stomach, Heart, Soleus, Mandibular lymph node, Mesenteric lymph node, lung, Spleen, Thymus, Bone marrow, Brain.

161 SDE probesets were expressed in a single tissue, i.e. they were not expressed with or without sex bias in any other tissue. The corresponding genes likely carry out organ-specific, sex biased functions.They may therefore be of particular interest for further investigation, and can be identified in appendix 1.

Most SDE probesets were also expressed without sex bias in multiple other tissues. This indicates that sexually dimorphic expression of genes is, in most cases, tissue-specific.

Ubiquitously-expressed genes were less likely than other genes to be SDE (6.17% *vs* 17.1%). This concords with the common sense expectation that many are housekeeping genes with an essential role in both sexes.

**Figure 3:**
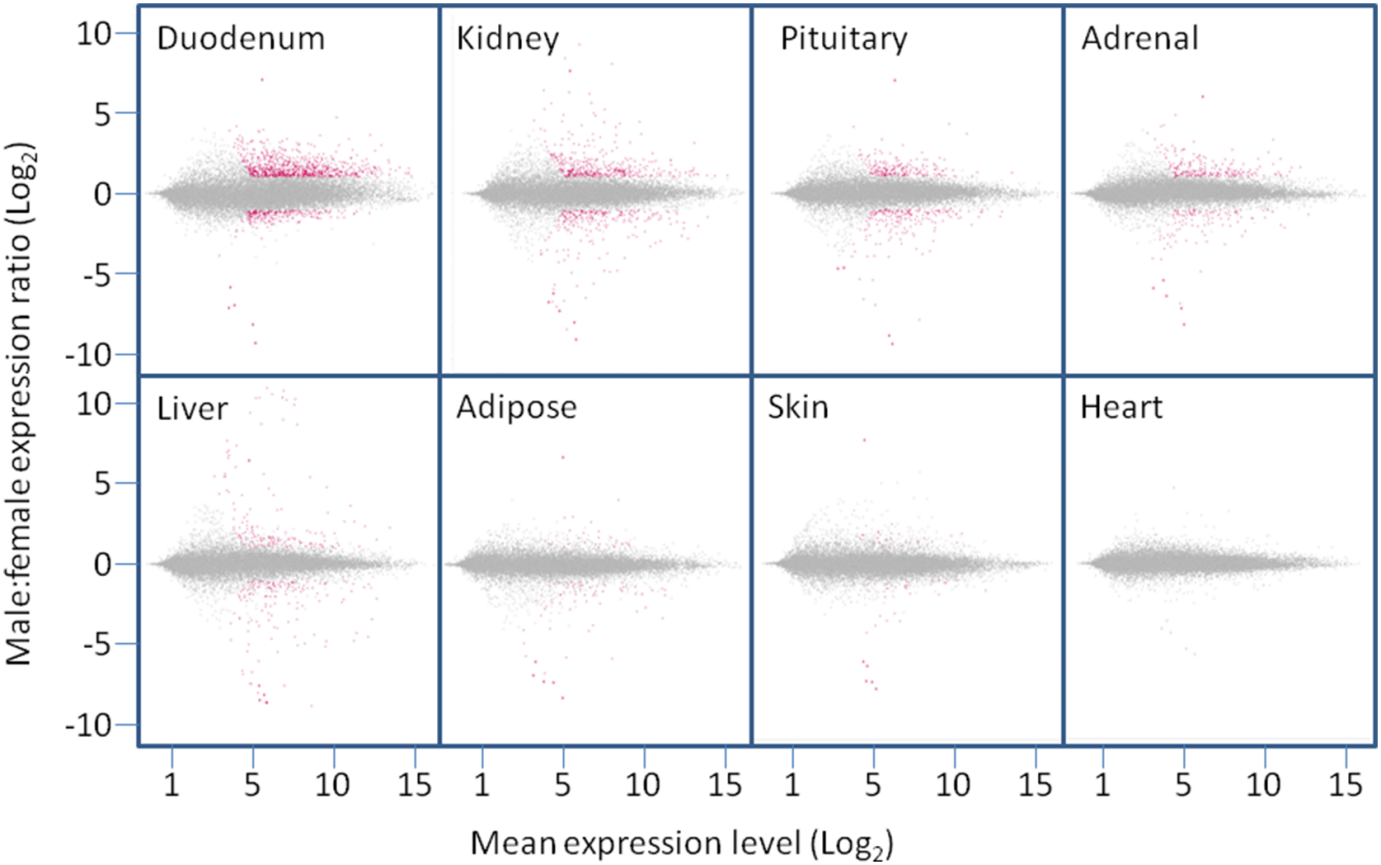
Mean Average (MA) plots for selected somatic rat tissues. The log_2_ of the M:F expression ratio is plotted against the log_2_ of the mean average (both sexes) expression level. Probesets meeting the criteria for sexually dimorphic expression in the specific tissue are coloured red, the remainder grey. Probesets that are SDE in more than 10 tissues (the six probesets representing Eif2s3y and Xist) are represented by larger markers. The male and female expression levels of these probesets are well conserved across tissues, even though differences between the sexes are not always statistically significant, for example in the heart.

To enable the distribution of SDE probesets in specific tissues to be better visualised, MA plots (log_2_ male:female expression ratio plotted against log_2_ mean expression intensity) [10] were generated, with examples shown in Fig. 3. These plots illustrate how the combined threshold, q-value and -fold change cutoffs together identify probesets that are robustly SDE in these representative tissues. Probesets differentially expressed in 10 or more tissues (i.e. those representing *Xist* and *Eif2s3y)* are represented by larger markers, and are seen at the extremes of the y-axis in each tissue. These probesets fail to be flagged as SDE in the heart since they do not achieve the necessary q-value, but nevertheless exhibit an expression level and sex ratio consistent with sexually-dimporphic expression. These plots illustrate how the distribution of SDE probesets differs between tissues; those in the duodenum mostly exhibit moderate -fold differences between the sexes, whilst larger differences are seen for liver and kidney -specific SDE probesets, where well-characterised genes with sex-specific expression are known to occur [11], [2].

### Analysis of SDE probesets in the duodenum

**Figure 4:**
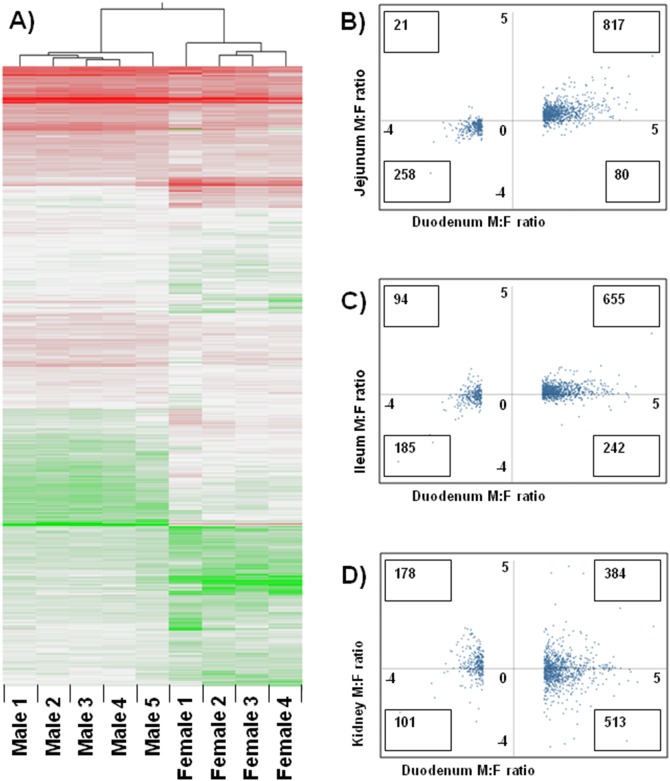
Evidence that SDE probesets in the duodenum are genuine. A) Hierarchical clustering shows that SDE probesets in the duodenum are consistently biased in individual animals according to sex, such that same-sex samples cluster, and the two sexes segregate. B) Probesets identified as SDE in the duodenum have a similar sex bias in the adjacent jejunum, even though the majority fail to pass the criteria for sexually dimorphic expression in the jejunum. C) They also show the same bias, albeit weaker, in the ileum, a nonadjacent section of the small intestine. D) By contrast, they show no correlated sex bias in the kidney.

We were struck by the high incidence of SDE probesets in the duodenum. Several approaches were used to rule out the possibility that these were artefactual. Firstly, gene expression plots were generated for every possible pair of duodenum samples. Consistently higher correlation coefficients were seen for same-sex pairs (0.860+/−0.09, mean +/−S.D.) than different-sex pairs (0.429+/−0.09) (Student’s T-test p-value <0.0001). Secondly, hierarchical clustering showed a clear clustering of same-sex duodenum samples, and separation between the sexes (Fig. 4a). Thirdly, probesets identified as SDE in the duodenum tended to have a similar but less pronounced dimorphic scew in the adjacent jejunum, less again in the nonadjacent ileum, and no similar dimorphism in an unrelated organ, the kidney (Fig. 4b–d). Together, these observations support the conclusion that the probesets identified as SDE in duodenum are genuinely SDE.

### Identification of SDE genes and networks

We hypothesized that multiple SDE genes within a tissue may function together to create a sexually dimorphic phenotype. We therefore sought to identify such networks in the liver and duodenum by way of example, using the Ingenuity Pathway Analysis (IPA) software.

**Figure 5:**
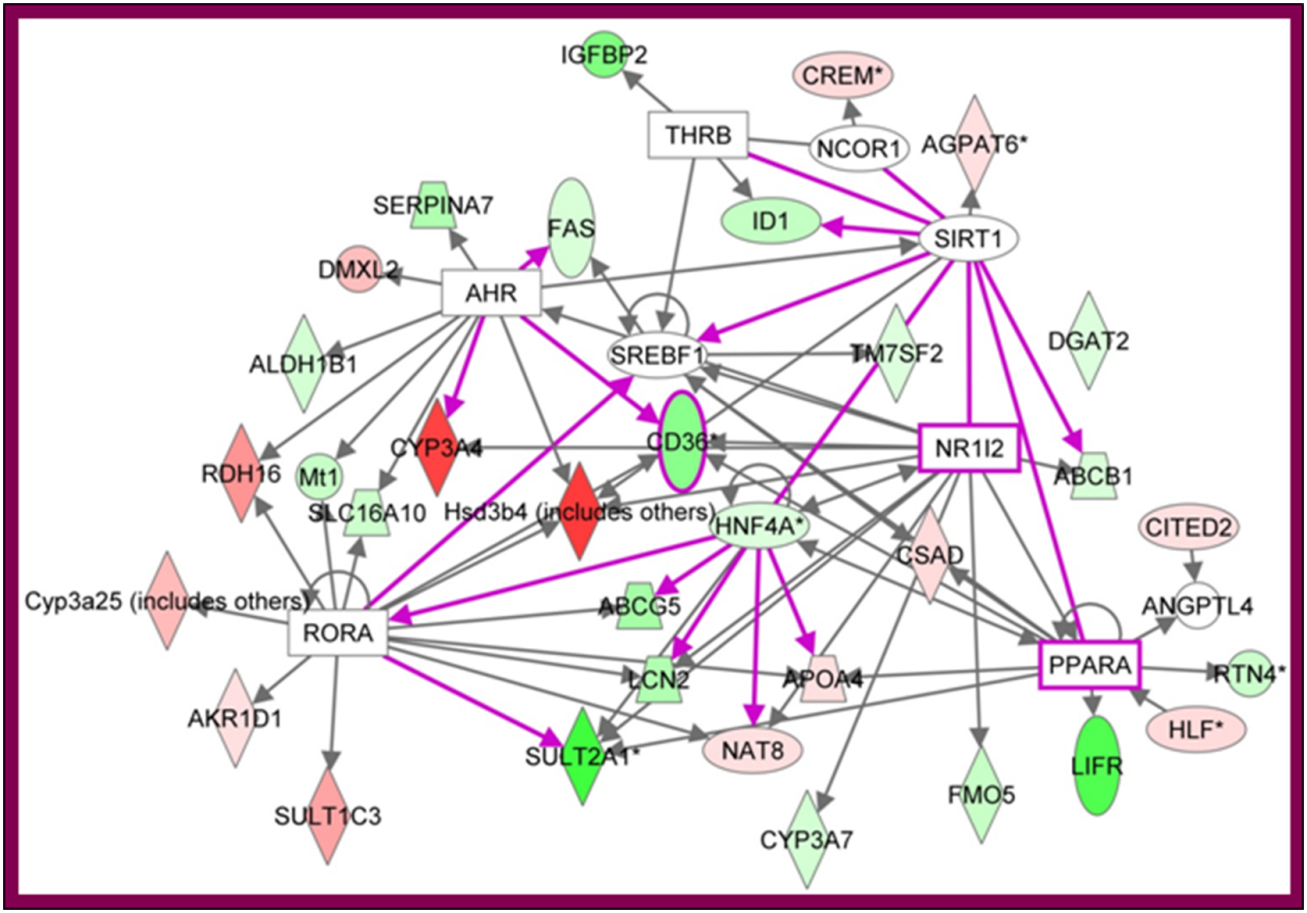
Ingenuity Pathway Analysis network based on SDE probesets in the liver. Numbers below each gene symbol indicate the mean log_2_–fold difference between male and female samples. Genes preferentially expressed in males are red, and in females green.

330 SDE probesets were identified in the liver, mapping to 271 genes in IPA. Notable molecules included multiple p450 CYP molecules, with *CYP2C9*, *CYP3A4*, *CYP2A12* and *CYP4A22* more highly expressed in the male, and *CYP2C12* more higly expressed in the female. Several other catabolic enzymes, including sulphotransferases and dehydrogenases were also SDE. Several female-biased genes associated with immune function were also noted, including *IRF1*, *IGIFBP*, *IL-13Ra1*, *IFNGR2*, and *LIFR*. The top two diseases associated with the genes were gastrointestinal disease and hepatic system disease (7 molecules each, p-value 7.33E-3-4.52E-2). The top three molecular/cellular functions were Lipid Metabolism (12 molecules, p-value 7.33E-3-4.52E-2), Molecular Transport (11 molecules, p-value 7.33E-3-4.52E-2) and small molecule biochemistry (13 molecules, p-value 7.33E-3-4.52E-2). The top three associated networks were Lipid Metabolism, Small Molecule Biochemistry, Molecular Transport; Lipid Metabolism, Small Molecule Biochemistry, Gastrointestinal Disease, and Drug Metabolism, Lipid Metabolism, Molecular Transport. The top network, extended to include other liver SDE genes, is shown in Fig. 5. This network indicates that HNF4A* is a key SDE gene, functionally associated with several others. The network also identifies *NR1/2* and *PPARA* as potential common regulatory nodes, although they are not SDE. The identification of these networks suggests that SDE genes may be regulated, and act in concert, in the liver. These networks may partly account for known differences between male and female liver function.

**Figure 6:**
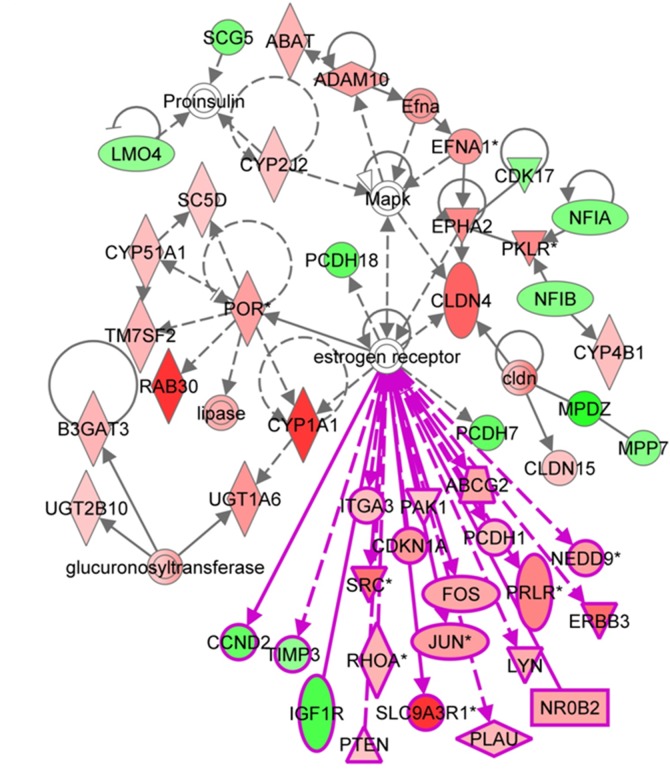
A network based on SDE probesets in the duodenum that is associated with the term ‘Lipid Metabolism, Small Molecule Biochemistry, Embryonic development, extended to show probesets associated with the ER.

1176 SDE probesets were identifified in the duodenum, mapping to 994 genes in IPA. These appeared to be quite functionally diverse. The top diseases associated with the genes were Gastrointestinal Disease (185 molecules, p-value 5.63E-8-5.73E-3) and Hepatic System Disease(152 molecules, p-value 5.63E-8-5.73E-3). The top molecular/cellular functions were Lipid Metabolism (198 molecules, p-value 2.92E-13-5.03E-3) Molecular Transport (257 molecules, p-value 2.92E-13-6.46E-3) and Small Molecule Biochemistry (233 molecules, p-value 2.92E-13-5.03E-3). The top four associated networks were 1) Embryonic Development, Nervous System Development and Function, Organ Development, 2) post-translational modification, Cancer, Organ Morphology, 3) molecular transport, Organismal injury and Abnormalities, Renal and Urological Disease, and 4) Lipid metabolism, Small Molecule Biochemistry, Embryonic Development. The latter network, extended to include other duodenum SDE genes, is shown in Fig. 6. This network reveals a complex network of SDE probesets in the duodenum. Notably, many SDE genes, predominantly with higher expression in female duodenum, were associated with the estrogen receptor, which was not SDE.

### Genes that are SDE in multiple tissues: opportunities for generating hypotheses

**Figure 7:**
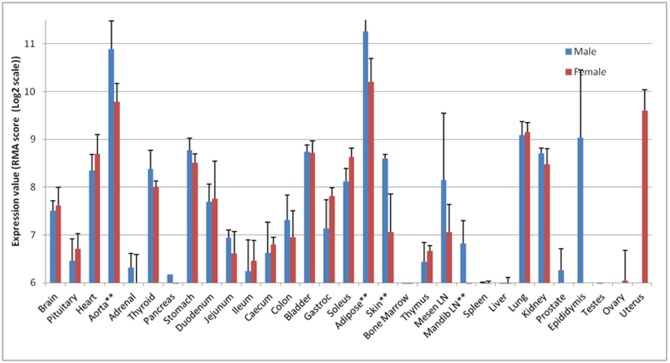
Expression of Pmepa (probeset 1389809_at) in male and female tissues. In starred (**) tissues, the criteria for sexually dimorphic probeset expression are met. A minimum expression value of 6.65 is required for a probeset to be considered as expressed.

We considered that the sexually dimorphic expression of genes in multiple tissues could reveal novel insights into their function, since it implies that those tissues have a commonality related to the SDE genes they share. *Pmepa1,* (Prostate Transmembrane Protein, Androgen Induced 1), significantly male-biased in the skin, aorta, and adipose, was considered as one example. (Fig. 7). Interestingly, *Pmepa1* is highly expressed in other tissues, e.g. the lung and the heart, but without sex bias. Further, it was only marginally expressed in some other tissues including the prostate, where it was first discovered, and was undetectable in the bone marrow, spleen and liver. It was highly expressed in the uterus. *Pmepa1* is a negative regulator of the androgen receptor [12], leading us to speculate that it may be most highly expressed in tissues where the androgen drive needs to be limited.

Of particular interest are instances where genes are oppositely biased in different tissues. *Prlr*, encoding the Prolactin Receptor, was found to be preferentially expressed in the male kidney and duodenum, and the female liver. It was highly expressed without sex bias in other tissues. Together, these observations reveal a complex pattern of *Prlr* expression, only partly described previously [13] and currently lacking proper explanation.

## Discussion

By transcript-profiling a broad range of tissues from male and female rats, we showed that approximately 14.5% of all expressed genes are SDE in at least one tissue in the rat. Most were SDE in a single tissue, but expressed in other tissues also, indicating that sexually dimorphic expression is determined primarily by the tissue rather than the gene. Overall, SDE genes were more often preferentially expressed in males than females, though the identification of possible sex-specific transcriptional regulators is beyond the scope of this paper.

Where probesets exhibited SDE in multiple tissues, opposite bias in different tissues was often seen. This has been noted for individual genes before, (e.g. AP-2 is expressed in different areas of the male vs female brain. [14]), but the scale of complexity reported here is greater than previously reported. Again, this complexity suggests that sexually dimorphic gene expression is largely tissue- rather than gene- specific. Here, we greatly extend the list of genes with such behaviour. This raises the challenge to understand the relevance for individual genes, and the underlying drivers of such behaviour.

*Eif2s3y* was conspicuous as the only gene that was ubiquitously expressed in all tissues, but exclusively in males. The female homologue *Eif2s3x* was also ubiquitously expressed, and was consistently more highly expressed in females, though it did not meet our relatively strict criteria for a SDE gene in all tissues. This is consistent with previous reports showing that *Eif2s3x* remains transcriptionally active on the condensed X chromosome in females, such that females may express more mRNA than males [15]. *Eif2sy* expression may functionally compensate for the lesser dosage of *Eif2s3x* in males. However, *Eif2s3y* is also known to be indispensable for spermatogenesis [16], suggesting it cannot be wholly substituted by *Eif2s3x*. Cross-tissue analysis reveals that the testes have amongst the lowest expression of eif2s3y, yet the highest ratio of *Eif2s3y:Eif2s3x*, owing to very low expression of *Eif2s3x*. This observation may be worthy of further exploration, perhaps indicating that the *Eif2s3y* gene product is dose-limiting in the testes, creating evolutionary pressure for the maintenance of the Y chromosome. Further Y-determined mechanisms may prevent the functional substitution of *Eif2s3x* for *Eif2s3y* in the process of spermatogenesis.

Two-way hierachical clustering was used to reveal possible structure in the distribution of SDE probesets that may not be apparent from simple numerical analysis. Clustering of tissues was weak, suggesting that each tissue had a largely individual complement of SDE genes. Clustering of probesets was also quite weak, although three large groups could be discerned of probesets that were either broadly male-biased, female-biased, or differently biased in the two large tissue groups. Further numerical analysis of SDE probeset similarity between tissues also failed to find stong groupings, though a greater-than-chance sharing of SDE probesets between the duodenum and kidney, and possibly the adrenal and pituitary, was seen.

The different incidences of SDE probesets between tissues was striking, ranging from 1176 in the duodenum to none (other than the six ubiquitous SDE probesets) in nearly half of the tissues surveyed. This suggests that the function of some tissues is much more gender-specific than that of others. This observation *per se* is not surprising; however, the identity of some tissues at either end of the spectrum was. For example, functional differences in the immune systems of males and females are well known [17], [18], and gene expression in the immune system can be affected by sex hormones. Nevertheless, immune tissues had relatively few SDE probesets. Similarly, great focus has been placed on gender differences in gene expression in the brain [5], [19], [20], though our study suggests these are numerically small at the whole-brain level. Localized differences in gene expression may however occur, but would be unobservable in whole-organ homogenates.

The duodenum had the largest number of SDE probesets, which we found surprising. One possible explanation is that sexual dimorphism in the duodenum may be secondary to sex differences in the gut microbiome, which have recently been shown to have far-reaching consequences on gut biology and susceptibility to disease [21].

The SDE genes in a tissue could be assembled into informative networks using Ingenuity Pathway Analysis. This identified *Hnf4a* as a key node in the liver, consistent with previous reports [22]. These differences are well known, and account for sexual dimorphism in hormone and drug metabolism by the liver [23], [24], [25].

The SDE genes in the duodenum were enriched in particular phenotypic descriptors. Interestingly, gastrointestinal disease was a top hit, suggesting that SDE genes could be important players in disease, perhaps playing a role in sex differences in disease susceptibility. We noted particularly the prevalence of SDE genes associated with the estrogen receptor (ERA), suggestive of a hormone dependency in SDE gene expression.

This study has identified a plethora of SDE genes, many with complex patterns of expression across multiple tissues. Individual genes may be expressed, non-expressed, male- and female biased, simultaneously in different tissues. Such behaviour suggests that the sexually dimorphic expression of genes is highly plastic, and is primarily dictated by the context of the tissue in which it is expressed rather than by intrinsic properties of the gene. SDE genes are likely to affect sexually dimorphic phenotypes, with implications for disease and drug susceptibility. The full set of SDE probesets identified in rat tissues is presented as a resource for further investigation.

## Supporting Information

S1 Table **Identity, distribution and expression properties of probesets that are sexually dimorphically expressed.** The incidence of sexually dimorphically expressed probesets across tissues is recorded in the matrix at the right hand side of the spreadsheet. The expression levels, fold-differences and Q values that contribute to the identification of SDE probesets are also given.
(XLSX)

S2 Table **Quality assessment of RNA isolated from rat tissues.**
(XLSX)
